# A prospective, multicenter trial of a long-term bioabsorbable mesh with Sepra technology in cohort of challenging laparoscopic ventral or incisional hernia repairs (ATLAS trial)

**DOI:** 10.1016/j.amsu.2021.103156

**Published:** 2021-12-06

**Authors:** William W. Hope, Adel G. El-Ghazzawy, Brad A. Winterstein, Jeffrey A. Blatnik, S. Scott Davis, Jacob A. Greenberg, Noel C. Sanchez, Eric M. Pauli, Daniel M. Tseng, Karl A. LeBlanc, Kurt E. Roberts, Curtis E. Bower, Eduardo Parra-Davila, J. Scott Roth, Corey R. Deeken, Eric F. Smith

**Affiliations:** a New Hanover Regional Medical Center, Department of Surgery, Wilmington, NC, United States; b Overlake Medical Center, Department of Surgery, Bellevue, WA, United States; c Methodist Hospital, Department of Surgery, Omaha, NE, United States; d Washington University, Department of Surgery, St. Louis, MO, United States; e Emory University, Department of Surgery, Atlanta, GA, United States; f University of Wisconsin, Department of Surgery, Madison, WI, United States; g Via-Christi Hospital, Department of Surgery, Wichita, KS, United States; h Penn State Hershey Medical Center, Department of Surgery, Hershey, PA, United States; i Legacy Health, Department of Surgery, Portland, OR, United States; j Our Lady of the Lake Regional Medical Center, Department of Surgery, Baton Rouge, LA, United States; k St. Francis Hospital, Department of Surgery, Hartford, CT, United States; l Carilion Clinic, Department of Surgery, Roanoke, VA, United States; m Celebration Health, Department of Surgery, Celebration, FL, United States; n University of Kentucky, Department of Surgery, Lexington, KY, United States; o Covalent Bio, LLC, St. Louis, MO, United States; p Georgetown Community Hospital, Department of Surgery, Georgetown, KY, United States

**Keywords:** Laparoscopic ventral hernia repair, Laparoscopic incisional hernia repair, Poly-4-hydroxybutyrate, Recurrence, Surgical site infection, Surgical site occurrence

## Abstract

**Background:**

This prospective, multicenter, single-arm, open-label study evaluated P4HB-ST mesh in laparoscopic ventral or incisional hernia repair (LVIHR) in patients with Class I (clean) wounds at high risk for Surgical Site Occurrence (SSO).

**Methods:**

Primary endpoint was SSO requiring intervention <45 days. Secondary endpoints included: surgical procedure time, length of stay, SSO >45 days, hernia recurrence, device-related adverse events, reoperation, and Quality of Life at 1, 3, 6, 12, 18, and 24-months.

**Results:**

120 patients (52.5% male), mean age of 55.0 ± 14.9 years, and BMI of 33.2 ± 4.5 kg/m^2^ received P4HB-ST mesh. Patient-reported comorbid conditions included: obesity (86.7%), active smoker (45.0%), COPD (5.0%), diabetes (16.7%), immunosuppression (2.5%), coronary artery disease (7.5%), chronic corticosteroid use (2.5%), hypoalbuminemia (0.8%), advanced age (10.0%), and renal insufficiency (0.8%). Hernia types were primary ventral (44.2%), primary incisional (37.5%), recurrent ventral (5.8%), and recurrent incisional (12.5%). Patients underwent LVIHR in laparoscopic (55.8%) or robotic-assisted cases (44.2%), mean defect size 15.7 ± 28.3 cm^2^, mean procedure time 85.9 ± 43.0 min, and mean length of stay 1.0 ± 1.4 days. There were no SSOs requiring intervention beyond 45 days, n = 38 (31.7%) recurrences, n = 22 (18.3%) reoperations, and n = 2 (1.7%) device-related adverse events (excluding recurrence).

**Conclusion:**

P4HB-ST mesh demonstrated low rates of SSO and device-related complications, with improved quality of life scores, and reoperation rate comparable to other published studies. Recurrence rate was higher than expected at 31.7%. However, when analyzed by hernia defect size, recurrence was disproportionately high in defects ≥7.1 cm^2^ (43.3%) compared to defects <7.1 cm^2^ (18.6%). Thus, in LVIHR, P4HB-ST may be better suited for small defects. Caution is warranted when utilizing P4HB-ST in laparoscopic IPOM repair of larger defects until additional studies can further investigate outcomes.

## Highlights

• Prospective, multicenter, single-arm, open-label study of P4HB-ST mesh LVIHR.
• Class I (clean) wounds at high risk for Surgical Site Occurrence (SSO).
• P4HB-ST exhibited few SSO and device-related complications, and improved QoL.
• Recurrence rate was higher than expected, especially in large defects (≥7.1 cm^2^).
• In LVIHR, P4HB-ST may be better suited for small defects (<7.1 cm^2^).

## 1 Introduction

Biomaterials have been utilized to repair incisional hernias for more than half a century [1]. Permanent synthetic materials were among the first such biomaterials and continue to be the gold standard for incisional hernia repair [2]. With the advent of laparoscopic surgery, intraabdominal mesh placement became routine, necessitating the development of new devices commonly described as “composites” [3,4]. Devices in this broad category are comprised of a structural mesh combined with a barrier layer that is intended to minimize tissue attachment between the abdominal viscera and the mesh. Both the underlying structural mesh and the barrier can be comprised of a variety of materials, including permanent synthetic polymers, biological tissue-derived materials, and absorbable polymers [[3], [4], [5]].

As described by the Deeken & Lake Mesh Classification System in a recent review article, a multitude of possible combinations exist, with 7 sub-categories of “barrier” devices encompassing more than 40 designs [5]. One particularly novel design is P4HB-ST mesh (Phasix™ ST Mesh, C. R. Bard/Davol, Inc., Warwick, RI), which represents the only fully absorbable barrier mesh construct. P4HB-ST mesh is comprised of an absorbable polymer scaffold of poly-4-hydroxybutyrate (P4HB) combined with an absorbable hydrogel barrier layer (ST) of sodium hyaluronate, carboxymethylcellulose, and polyethylene glycol [6]. The barrier is absorbed over a period of approximately 30 days, while the underlying P4HB scaffold is absorbed by 12–18 months [6]. The predictable absorption of the components results in a gradual transfer of load from the mesh back to the remodeled abdominal wall. The medium-term absorption profile of P4HB meshes (Phasix™ Mesh & Phasix™ ST Mesh: 12–18 months) [6,7] provides support to the repair site longer than short-term absorbable materials such as glycolide:lactide (Vicryl®: 2–3 months) [8] and polyglycolic acid:trimethylene carbonate (Bio-A®: 6–7 months) [9]. This is an important benefit since a mesh that is absorbed before newly deposited host collagen has matured may result in a hernia recurrence due to a lack of mechanical support at the repair site.

There have been favorable long-term outcomes reported with P4HB mesh in open cases [10,11]. However, there have been no reports on clinical outcomes when used in a minimally invasive manner. The purpose of this study is to evaluate P4HB-ST mesh in minimally invasive hernia repair in Class I (clean) wounds at high risk for SSO, and represents the first clinical trial of P4HB-ST. Patients at high risk for SSO were chosen to allow comparison with prior studies of P4HB mesh without the ST layer.

## 2 Materials and methods

### 2.1 Study design

The objective of this prospective, multicenter, single-arm, open-label study (ClinicalTrials.gov/NCT02712398) was to assess the safety, performance, and effectiveness of P4HB-ST mesh (Phasix™ ST Mesh, C. R. Bard, Inc., Warwick, RI) in laparoscopic/robotic ventral or incisional hernia repair (LVIHR) in a cohort at high risk for surgical site occurrences (SSO). Patients were considered at high risk for SSO with one or more of the following comorbid conditions: body mass index (BMI) between 30 and 40 kg/m^2^(inclusive), active smokers, chronic obstructive pulmonary disease (COPD), diabetes mellitus, immunosuppression, coronary artery disease, chronic corticosteroid use (>6 months systemic use), hypo-albuminemia (preoperative serum albumin <3.4 g/dL), advanced age (≥75 years), or renal insufficiency (serum creatinine concentration ≥2.5 mg/dL). The study was designed to treat 120 patients at approximately 16 sites throughout the United States. Investigators were chosen due to experience with minimally invasive hernia repair techniques. Specific training was not required based on the similarity in technique required for P4HB-ST mesh compared to other meshes. The protocol was approved by the Institutional Review Board (IRB) at each institution, and all subjects provided informed consent prior to enrollment. Recruitment occurred between May 4, 2016 and November 27, 2017 through the surgical offices of the Investigators according to the eligibility criteria.

### 2.2 Inclusion/exclusion criteria

Patients with a diagnosis of ventral or abdominal incisional hernia with a planned laparoscopic/robotic surgical repair with defect closure were screened for study eligibility against the study protocol inclusion and exclusion criteria. Patients were included in the study if they were 18 years of age or older, met the criteria for a Class I (clean) wound as defined by the CDC [12], had one or more of the comorbidities listed above, were willing to undergo laparoscopic hernia repair with intraabdominal placement of P4HB-ST mesh, and provided written informed consent.

Patients were excluded if they had four or more previous hernia repairs of the index hernia, a hernia defect greater than 350 cm^2^, existing mesh in the affected area that could not be removed, permanent mesh adjacent to the current hernia, planned preperitoneal approach, a known collagen disorder, peritonitis, on or may be placed on chemotherapy medications during the study period, BMI >40 kg/m^2^, cirrhosis of the liver and/or ascites, American Society of Anesthesiology Class 4 or 5, life expectancy <2 years, surgical wound classified as Class II (clean-contaminated), Class III (contaminated), or Class IV (dirty-contaminated) as defined by the CDC [12], active or latent systemic infection, contraindication to placement of mesh, planned bridge repair, pregnant or plans to become pregnant during the study period, enrolled in another interventional clinical study within the last 30 days, part of the study site personnel directly involved with the study, known allergy to the test device or component materials, or any condition that, in the opinion of the investigator, would preclude the use of the study device or preclude the patient from completing the follow-up requirements.

### 2.3 Surgical technique

All patients underwent laparoscopic or robotic ventral hernia repair with preoperative antibiotics administered according to hospital protocol. Intraoperative exclusion criteria (i.e., hernia >350 cm^2^, Class II, III, or IV wounds, latent or systemic infection, peritonitis, and bridge repair technique) were evaluated and recorded, and patients were screen-failed when applicable. The hernia defect was closed by reapproximating the fascia, including myofascial release (MR), if needed. Bridged repairs were not allowed according to the protocol, and the method of fascial closure was left up to the individual investigators. Patients that met the inclusion criteria received intraabdominal placement of P4HB-ST mesh. The prosthesis was positioned with its edges extending beyond the margins of the defect by at least 5 cm, and the coated side was oriented against the bowel. Fixation devices were applied around the periphery of the mesh at approximately 1–2 cm intervals, and the trocar sites were closed with sutures and/or staples. Wounds were dressed with sterile occlusive dressings, and postoperative care was performed consistent with surgeon practice at each site.

### 2.4 Data collection

Surgical details, including procedure date, start/stop times, hernia defect size, mesh size, repair technique, concomitant procedures, adverse events, and procedure-related complications were documented. Follow-up visits were scheduled for 1, 3, 6, 12, 18, and 24 months postoperatively. At each visit, Quality of Life assessments, device-related adverse events, hernia recurrence, concomitant pain medication usage, and surgical complications were documented.

### 2.5 Study endpoints

The primary endpoint of the study was Surgical Site Occurrence (SSO) requiring intervention within 45 days postimplantation, including Surgical Site Infection (SSI), seroma, hematoma, wound dehiscence, skin necrosis, mesh infection and fistula. This timeframe was chosen as most SSOs occur in the early postoperative period, and it was desirable to assess SSOs during the period in which the ST barrier was still intact. Secondary endpoints included: surgical procedure time, length of stay, SSO >45 days postimplantation, hernia recurrence rate, device-related adverse events, rate of reoperation of the index hernia repair, and Quality of Life assessments (Visual Analog Scale for pain, Carolinas Comfort Scale®, and SF-12v2®), assessed at 1, 3, 6, 12, 18, and 24-months.

### 2.6 Statistical analysis

GraphPad Prism 6.01 statistical software was utilized to generate descriptive statistics. Frequency counts and percentages are reported for categorical variables, while mean and standard deviation are reported for continuous variables. Hernia recurrence is reported via Kaplan-Meier estimates. Quality of life assessments were evaluated for statistical significance between baseline values and 24-month values using an unpaired, two-tailed *t*-test with Welch's correction (p < 0.05 statistically significant). This work complies with the STROCSS criteria (Strengthening the Reporting of Cohort Studies in Surgery) [13].

## 3 Results

### 3.1 Patient demographics

**Fig. 1:**
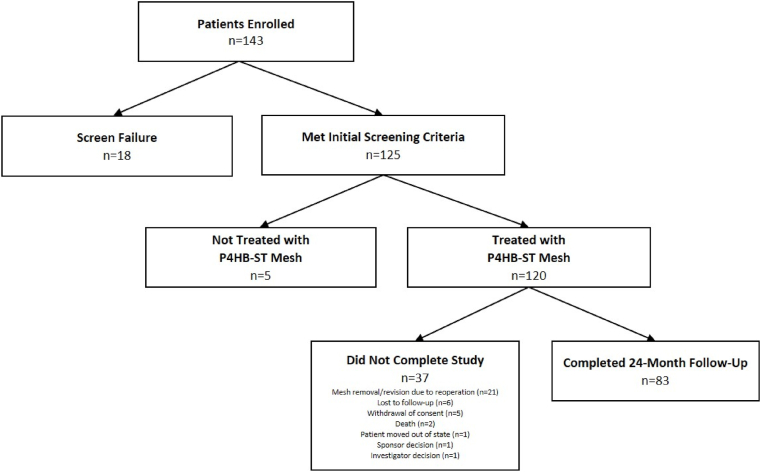
Flow of patients throughout the study period.

As shown in Fig. 1, a total of n = 143 patients were enrolled in the trial. Eighteen (n = 18) patients were excluded after the screening process, while n = 125 met all of the initial screening criteria. Of those n = 125 patients, a total of n = 120 patients were ultimately implanted with P4HB-ST mesh. As shown in Table 1, the patients had a mean age of 55.0 ± 14.9 years and BMI of 33.2 ± 4.5 kg/m^2^. Slightly more than half of the patients were male (n = 63, 52.5%), and the majority were White (n = 110, 91.7%). A small minority of patients experienced a prior repair of the index hernia (n = 22, 18.3%). Patient-reported comorbid conditions included: obesity (104/120, 86.7%), active smoker (54/120, 45.0%), diabetes (20/120, 16.7%), advanced age (12/120, 10.0%), coronary artery disease (9/120, 7.5%), COPD (6/120, 5.0%), immunosuppression (3/120, 2.5%), chronic corticosteroid use (3/120, 2.5%), hypoalbuminemia (1/120, 0.8%), and renal insufficiency (1/120, 0.8%). The majority of patients had 1 or 2 comorbidities (n = 104, 86.7%), with a minority reporting 3 or more comorbidities (n = 16, 13.3%).

**Table 1:** Preoperative data: Patient demographics and hernia data.

Patients treated with P4HB-ST mesh, n	120
**Patients with 24-month follow-up,** n (%)	83 (69.2%)
**Sex**	
Male, n (%)	63 (52.5%)
Female, n (%)	57 (47.5%)
**Race**	
Asian, n (%)	1 (0.8%)
Black, n (%)	7 (5.8%)
White, n (%)	110 (91.7%)
Biracial, n (%)	1 (0.8%)
Not reported, n (%)	1 (0.8%)
**Age (years),** mean ± SD	55.0 ± 14.9
**Body mass index,** kg/m^2^ (mean ± SD)	33.2 ± 4.5
**Patients with prior repairs to index hernia**	
0 prior repairs, n (%)	98 (81.7%)
1 prior repairs, n (%)	19 (15.8%)
2 prior repairs, n (%)	2 (1.7%)
3 prior repairs, n (%)	1 (0.8%)
**Number of Comorbidities**	
1 Comorbidity, n (%)	48 (40.0%)
2 Comorbidities, n (%)	56 (46.7%)
3 Comorbidities, n (%)	11 (9.2%)
4 Comorbidities, n (%)	5 (4.2%)
**Comorbidities**	
Obesity, n (%)	104 (86.7%)
Active smoker, n (%)	54 (45.0%)
Diabetes, n (%)	20 (16.7%)
Advanced age, n (%)	12 (10.0%)
Coronary artery disease, n (%)	9 (7.5%)
COPD, n (%)	6 (5.0%)
Immunosuppressed, n (%)	3 (2.5%)
Chronic corticosteroid use, n (%)	3 (2.5%)
Hypoalbuminemia, n (%)	1 (0.8%)
Renal insufficiency, n (%)	1 (0.8%)
**Hernia Diagnosis**	
Primary ventral, n (%)	53 (44.2%)
Primary incisional, n (%)	45 (37.5%)
Recurrent ventral, n (%)	7 (5.8%)
Recurrent incisional, n (%)	15 (12.5%)
**Hernia Location**	
Umbilical, n (%)	92 (76.7%)
Epigastric, n (%)	24 (20.0%)
Infraumbilical, n (%)	13 (10.8%)
Suprapubic, n (%)	6 (5.0%)
Subxiphoid, n (%)	4 (3.3%)
Other, n (%)	4 (3.3%)

### 3.2 Preoperative data

As shown in Table 1, hernia types included primary ventral (53/120, 44.2%), primary incisional (45/120, 37.5%), recurrent ventral (7/120, 5.8%), and recurrent incisional (15/120, 12.5%). The majority of hernias were umbilical (n = 92, 76.7%), with epigastric, infraumbilical, suprapubic, subxiphoid, and “other” comprising the remaining hernia locations.

### 3.3 Perioperative data

Patients underwent minimally invasive hernia repair via laparoscopic (67/120, 55.8%) or robotic-assisted technique (53/120, 44.2%) with a mean defect size of 4.6 ± 3.8 cm length, 3.3 ± 2.5 cm width, and 15.7 ± 28.3 cm^2^ area (Table 2). A number of defects were described as “Swiss cheese” (n = 22, 18.3%). An intraabdominal technique without myofascial release (MR) was utilized in the majority of patients (n = 118, 98.3%). However, in n = 2 (1.7%) patients, MR was performed via endoscopic/minimally invasive (MIS) technique. All defects were reinforced with P4HB-ST mesh with a mean length of 16.0 ± 4.3 cm, width of 14.3 ± 3.3 cm, and area of 182.6 ± 74.9 cm^2^. Fixation devices were spaced an average of 1.2 ± 1.2 cm around the periphery of the mesh. A variety of fixation devices were used, ranging from suture, mechanical fixation, or a combination of suture and mechanical fixation. No deliberately bridged repairs were performed. However, the investigators closed the fascia using their own selected technique, and a variety of fascial closure techniques were employed. The average surgical procedure time was 85.9 ± 43.0 min (mean ± standard deviation, Table 3), and the majority of the patients did not require a drain (n = 115, 95.8%). When a drain was placed, the average drain duration was 15.7 ± 9.9 days (mean ± standard deviation). The average length of stay was 1.0 ± 1.4 days (mean ± standard deviation, Table 3).

**Table 2:** Perioperative data: Mesh/defect sizes and surgical technique (MR = myofascial release).

Type of Procedure	
Laparoscopic only, n (%)	67 (55.8%)
Robotic-assisted, n (%)	53 (44.2%)
**Surgical Technique**	
Intraabdominal without MR, n (%)	118 (98.3%)
Intraabdominal with MR, n (%)	2 (1.7%)
**Defect**	
Length, cm (mean ± SD)	4.6±3.8
Width, cm (mean ± SD)	3.3±2.5
Area, cm^2^ (mean ± SD)	15.7±28.3
Swiss Cheese Defect, n (%)	22 (18.3%)
**Mesh**	
Length, cm (mean ± SD)	16.0±4.3
Width, cm (mean ± SD)	14.3±3.3
Area, cm^2^ (mean ± SD)	182.6±74.9
Area Ratio, mean ± SD	34.6±35.6
**Fixation**	
Fixation spacing, cm (mean ± SD)	1.2±1.2
Suture & Mechanical, n (%)	52 (43.3%)
Suture only, n (%)	35 (29.2%)
Mechanical only, n (%)	33 (27.5%)
**Drains**	
0 drains, n (%)	115 (95.8%)
1 drain, n (%)	2 (1.7%)
≥2 drains, n (%)	3 (2.5%)

**Table 3:** Primary & secondary study endpoints (p < 0.05 compared to Baseline).

Study Endpoints:	Primary: SSO ≤45-day	Secondary: SSO >45-day
**SSI,** n (%)	0 (0.0%)	0 (0.0%)
**Seroma,** n (%)	0 (0.0%)	0 (0.0%)
**Hematoma,** n (%)	1 (0.8%)	0 (0.0%)
**Wound Dehiscence,** n (%)	0 (0.0%)	0 (0.0%)
**Skin Necrosis,** n (%)	0 (0.0%)	0 (0.0%)
**Mesh Infection,** n (%)	0 (0.0%)	0 (0.0%)
**Fistula,** n (%)	0 (0.0%)	0 (0.0%)
**Other Secondary Endpoints:**		
**Surgical procedure time,** min (mean ± SD)	85.9 ± 43.0	
**Length of stay,** days (mean ± SD)	1.0 ± 1.4	
**Hernia recurrence rate,** n (%)	38 (31.7%)	
**Reoperation rate,** n (%)	22 (18.3%)	
**Reason for Reoperation**		
*Hernia recurrence, n*	22	
*Additional procedures, n*	2	
**Device-related adverse events, n (%)** (excluding recurrence)	2 (1.7%)	
*Small bowel obstruction, n*	1	
*Abdominal pain, n*	1	
	***Baseline***	**24 months**
**Visual Analog Scale – Pain,** cm (mean ± SD)	1.99 ± 2.4	0.60 ± 1.5*
**Carolinas Comfort Scale® – Total Score** (mean ± SD)	1.15 ± 1.1	0.17 ± 0.4*
**SF-12® Physical Component Score** (mean ± SD)	44.4 ± 9.6	48.0 ± 9.7*
**SF-12® Mental Component Score** (mean ± SD)	53.5 ± 9.5	52.3 ± 9.6

### 3.4 Study endpoints

**Fig. 2:**
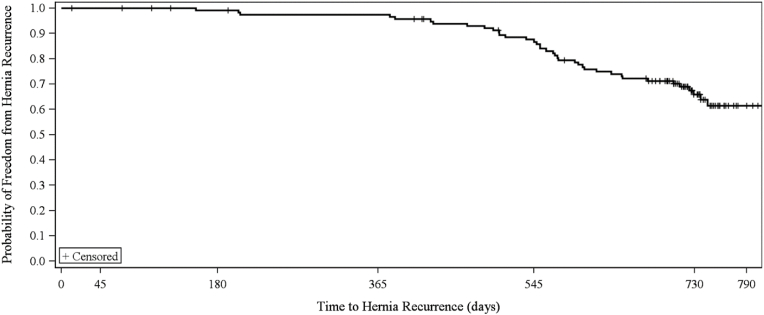
Kaplan-Meier curve for hernia recurrence.

A total of n = 83 (69.2%) patients completed the 24-month follow-up visit (Fig. 1 & Table 1). The majority of the n = 37 patients who did not complete the study had the mesh removed or revised during reoperation (n = 21), excluding those patients from later follow-up. A small number of patients were lost to follow-up (n = 6), withdrew consent (n = 5), died (n = 2), moved out of state (n = 1), or were removed from the study due to sponsor (n = 1) or investigator (n = 1) decision. The patient removed due to sponsor decision involved a mesh that was cut through, and the patient removed due to investigator decision was placed on hospice during the study period. As shown in Fig. 2 and Table 3, n = 38 (31.7%) patients experienced a hernia recurrence, n = 22 (18.3%) required a reoperation, n = 2 (1.7%) reported device-related adverse events (excluding recurrence), and n = 8 (6.7%) had the P4HB-ST mesh explanted at the time of reoperation. In half of the recurrences (n = 19; 50.0%), the mesh was fixated with a combination of suture and mechanical fixation, while the remaining n = 12 (31.6%) and n = 7 (18.4%) were fixated with only mechanical or suture fixation, respectively.

**Fig. 3:**
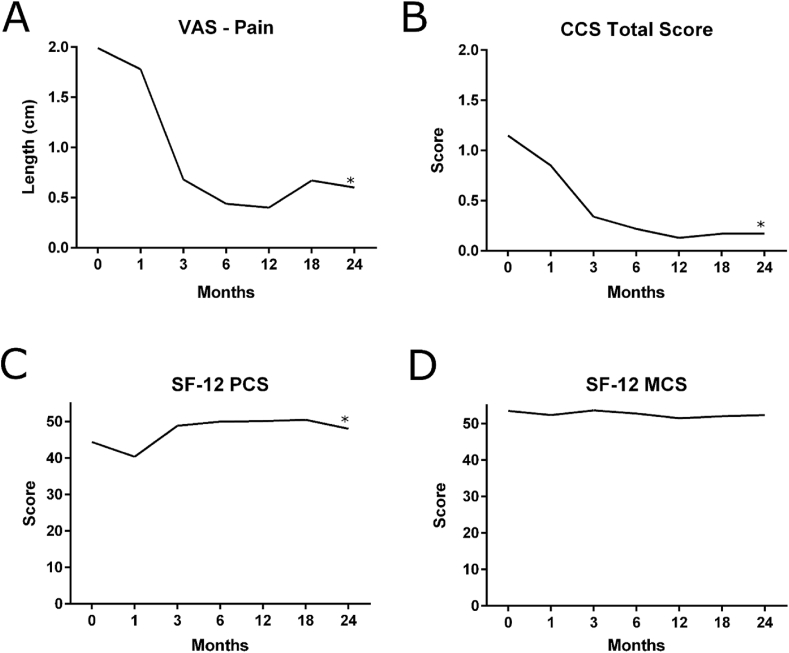
Quality of life assessments: **A)** Visual Analog Scale (VAS) for Pain (mean); **B)** Carolinas Comfort Scale® (CCS) – Total Score (mean); **C)** SF-12v2® Physical Component Score (PCS) (mean); **D)** SF-12v2® Mental Component Score (MCS) (mean); *p < 0.05 (Baseline vs. 24 months*)*.

Visual Analog Scores (VAS) for pain decreased significantly from 1.99 ± 2.4 prior to surgery (baseline) to 0.60 ± 1.5 at 24-months postimplantation (p < 0.05). Carolinas Comfort Scale® – Total Score decreased significantly from 1.15 ± 1.1 prior to surgery (baseline) to 0.17 ± 0.4 at 24-months postimplantation (p < 0.05). For the SF-12v2® scores, the Physical Component Score increased significantly from 44.4 ± 9.6 prior to surgery (baseline) to 48.0 ± 9.7 at 24-months postimplantation (p < 0.05), while the Mental Component Score remained unchanged from baseline (53.5 ± 9.5) to 24-month postimplantation (52.3 ± 9.6; p > 0.05). Quality of life assessment values for all intermediate time points are shown in Fig. 3.

A single SSO was reported within the first 45 days postimplantation (n = 1 hematoma, 0.8%; Table 3). The Kaplan-Meier estimate of SSO at 45-days follow-up was 0.8% (95% CI: 0.1%, 5.8%). There were no SSOs requiring intervention >45 days postimplantation (0/120, 0.0%). Hernia-related complications were graded according to the Clavien-Dindo system and are depicted in Supplementary Table 1 [14].

## 4 Discussion

This prospective, multicenter, single-arm, open-label study evaluated P4HB-ST mesh in LIVHR in patients with Class I (clean) wounds at high risk for SSO and represents the first clinical trial associated with this material in LIVHR. There is a previous trial reporting positive outcomes with the use of P4HB-ST at the hiatus during laparoscopic paraesophageal hernia repair [15], as well as favorable long-term data on the use of P4HB in the retrorectus space [10]. There are several interesting findings in our study. As expected, there was a low rate of SSO and device-related complications, with improvement in quality of life scores when utilizing P4HB-ST mesh in a minimally invasive approach to LIVHR. Similar outcomes have been documented in other large series with long-term follow-up [16]. The 31.7% hernia recurrence rate observed in this study was higher than previously reported 17.9% and 11% for open VHR with P4HB mesh [10,11]. However, recurrence in LIVHR is notoriously variable, ranging from 1% up to 29% in other series [[17], [18], [19], [20], [21]], with a variety of mesh types and patient factors contributing to these outcomes.

It is a common tenet in open ventral hernia repair that mesh reinforcement of fascial closure results in the best long-term results compared to a bridged repair [22,23]. Closure of fascia in minimally invasive surgery has been a recent topic of debate, as initially laparoscopic repairs were bridged repairs [16]. Recently, several studies have reported improvement in outcomes in patients undergoing defect closure in laparoscopic and robotic ventral hernia repair [[24], [25], [26]]. One of the inclusion criteria for this trial was fascial closure. As there have been good long-term outcomes associated with the P4HB material in open ventral hernia with low recurrence rates [10,11], it is possible that the higher recurrence rate in this trial was related to the nuances of fascial closure in minimally invasive repair compared to open repair. It should also be acknowledged that meshes were placed in the retrorectus or onlay position in studies utilizing an open technique, which is a different tissue plane than the current study. Tissue plane and fascial closure technique may both play a role.

One of the issues with fascial closure for laparoscopic ventral hernia repair is that suturing can be technically challenging, especially with large defects under tension. Techniques have been described to facilitate fascial closure in LIVHR, including the so called “shoelacing” technique [27]. Recently, robotic technology has been touted to improve the ability to close fascial defects in minimally invasive hernia repair compared to laparoscopic repair [26]. While there are some adjuncts that are improving the ability to close fascial defects in a minimally invasive manner, it is likely that this closure may not be as robust as when done open under direct visualization, and perhaps this is why there is some discrepancy in the recurrence rates.

Furthermore, when a fully absorbable material such as P4HB-ST is utilized to repair a hernia defect, effective fascial closure is critical. Slightly more than half of the patients in this study received barbed suture to close the fascia (n = 66; 55%; *data not shown*). Out of a total of n = 38 recurrences observed in this trial, 61% (n = 23; *data not shown*) occurred when barbed suture was used to close the fascia. Many types of barbed sutures have not been indicated for fascial closure. Compared to traditional, smooth sutures, the diameter of a barbed suture is reduced and the core weakened when the barbs are cut into the suture during the manufacturing process [28]. If barbed sutures are used for fascial closure and prematurely release from the fascia, the implanted P4HB-ST mesh will essentially function as a bridged repair. Other fully absorbable materials such as biological tissue-derived meshes have exhibited extremely high recurrence rates, reaching 80% in some series, when utilized to bridge a hernia defect [29]. Similar results would be expected for a fully absorbable synthetic material such as P4HB-ST mesh under bridging conditions, and the Instructions for Use for P4HB-ST mesh clearly warn against this practice [6].

Hernia defect area may also play a role in determining clinical outcomes such as recurrence. In this study, the majority of the defects were small with a median area of 7.1 cm^2^. When recurrence rate was analyzed at Day 730, defects <7.1 cm^2^ had a Kaplan-Meier hernia recurrence rate of 18.6%, regardless of whether barbed or smooth sutures were utilized for fascial closure. Recurrence rates were significantly and disproportionately higher in defects ≥7.1 cm^2^ (43.3%; p = 0.019). Thus, large defects may not be ideal for P4HB-ST when laparoscopic repair is planned, and suture selection is critical. If the abdominal fascia is closed with barbed suture and later fails, the repair will become a bridged repair, leading to a high likelihood of recurrence. Interestingly, the results of this study showed that suture selection is less impactful in small defects, allowing for more flexibility with the use of fully absorbable materials in small defects. P4HB-ST exhibited a K-M recurrence rate of 18.6% at Day 730 in defects <7.1 cm^2^, which is comparable to other long-term studies of both fully absorbable and permanent meshes [10,21,30,31]. Thus, P4HB-ST may be better suited for small defects.

## 5 Conclusions

P4HB-ST mesh demonstrated low rates of SSO and device-related complications, with improved quality of life scores, and reoperation rate comparable to other published studies [10,11,[19], [20], [21]]. Recurrence rate was higher than expected at 31.7%. However, when analyzed by hernia defect size, recurrence was disproportionately high in defects ≥7.1 cm^2^ (43.3%) compared to defects <7.1 cm^2^ (18.6%). Thus, in LVIHR, P4HB-ST may be better suited for small defects. Caution is warranted when utilizing P4HB-ST in laparoscopic IPOM repair of larger defects until additional studies can further investigate outcomes.

## Ethical approval

The protocol was approved by the Institutional Review Board (IRB) at each institution, and all subjects provided informed consent prior to enrollment.

## Funding

This study was sponsored by C. R. Bard, Inc. (Davol), Warwick, RI. (Bard has joined Becton Dickinson (BD).) Authors were reimbursed for expenses related to the conduct of the study.

## Author contribution

Dr. Hope – study concept or design, data collection, data analysis/interpretation, writing the paper. Dr. El-Ghazzawy – data collection, data analysis/interpretation, writing the paper. Dr. Winterstein – data collection, data analysis/interpretation, writing the paper. Dr. Blatnik – data collection, data analysis/interpretation, writing the paper. Dr. Davis – data collection, data analysis/interpretation, writing the paper. Dr. Greenberg – data collection, data analysis/interpretation, writing the paper. Dr. Sanchez – data collection, data analysis/interpretation, writing the paper. Dr. Pauli – data collection, data analysis/interpretation, writing the paper. Dr. Tseng – data collection, data analysis/interpretation, writing the paper. Dr. LeBlanc – data collection, data analysis/interpretation, writing the paper. Dr. Roberts – data collection, data analysis/interpretation, writing the paper. Dr. Bower – data collection, data analysis/interpretation, writing the paper. Dr. Parra-Davila – data collection, data analysis/interpretation, writing the paper. Dr. Roth – data collection, data analysis/interpretation, writing the paper. Dr. Deeken – data analysis/interpretation, writing the paper. Dr. Smith – data collection, data analysis/interpretation, writing the paper.

## Registration of research studies

Name of the registry: Clinical Trials.gov.

Unique Identifying number or registration ID: NCT02712398.

Hyperlink to your specific registration (must be publicly accessible and will be checked): https://clinicaltrials.gov/ct2/show/NCT02712398?term=02712398&draw=2&rank=1.

## Guarantor

William W. Hope, MD, FACS, 1725 New Hanover Medical Park Drive, Wilmington, NC 28401, Phone: (910) 662–9300, Fax: (910) 662–9301, Email: William.Hope@nhrmc.org.

## Provenance and peer review

Not commissioned, externally peer-reviewed.

## Declaration of competing interest

Drs. Davis, Tseng, Winterstein, Roberts, and El-Ghazzawy have no financial disclosures or conflicts of interest.

Dr. Blatnik is a paid consultant for BD, Intuitive, and Surgimatrix and receives research support from Cook and Ethicon.

Dr. Pauli is a paid consultant for Boston Scientific, Actuated Biomedical, Baxter, Wells Fargo, Cook Biotech, CMR Surgical, Neptune Medical, Surgimatix, Boehringer Laboratories, Allergan, and Noah Medical, receives speaking or teaching honoraria from Becton Dickinson (BD), Medtronic, Ovesco, and Boston Scientific, and receives royalties from UpToDate and Springer.

Dr. Smith is a paid speaker/teacher for BD and Intuitive Surgical.

Dr. Bower receives honoraria from BD.

Dr. Sanchez is a paid consultant for BD.

Dr. LeBlanc is a paid speaker for BD, Intuitive, and W.L. Gore.

Dr. Roth reports an institutional grant from C.R. Bard, Inc./Davol/Becton Dickinson (BD) during the conduct of this study. Dr. Roth also reports consulting fees (Johnson & Johnson and C.R. Bard, Inc./Davol/Becton Dickinson), institutional grant (Advanced Medical Solutions), stock (Miromatrix), and speaking fees (Allergan) outside of the current work.

Dr. Hope reports consulting fees and research support from C.R. Bard, Inc./Davol/Becton Dickinson (BD) during the conduct of the study. Dr. Hope also reports consulting fees, honoraria, and research support (Intuitive, W.L. Gore, and Medtronic) outside of the current work and participates in the Surgeon Advisory Board for Mesh Suture and Deep Blue.

Dr. Greenberg reports a grant from C.R. Bard, Inc./Davol/Becton Dickinson (BD) during the conduct of the study. Dr. Greenberg also reports grants (Becton Dickinson (BD) and Medtronic), as well as course registration, travel, and lodging (Intuitive) outside of the current work.

Dr. Parra-Davila has no conflicts of interest to disclose related to the current study. Dr. Parra-Davila reports consulting/speaking fees (C.R. Bard, Inc./Davol/Becton Dickinson (BD), Intuitive, Johnson & Johnson, and Medtronic), research funds (C.R. Bard, Inc./Davol/Becton Dickinson (BD)), and proctoring (Intuitive and C.R. Bard, Inc./Davol/Becton Dickinson (BD)) outside of the current work.

Dr. Deeken reports consulting fees from C.R. Bard, Inc./Davol/Becton Dickinson (BD) during the conduct of the study. Dr. Deeken also reports consulting fees from C.R. Bard, Inc./Davol/Becton Dickinson (BD), Johnson & Johnson, Medtronic, SurgiMatrix, Tissium, Surgical Innovation Associates, Americas Hernia Society Quality Collaborative, Colorado Therapeutics, TelaBio, and Aran Biomedical outside the submitted work. In addition, Dr. Deeken is the owner of Covalent Bio, LLC and holds the following issued patents: 2009293001, 2334257, 2,334,257UK, 602009046407.8, 2,334,257FR, 16/043,849 and 2,737,542.
