# Supplementary material for: A prospective, multicenter trial of a long-term bioabsorbable mesh with Sepra technology in cohort of challenging laparoscopic ventral or incisional hernia repairs (ATLAS trial)
Source: Ann Med Surg (Lond). 2021 Dec 6;73:103156. doi: 10.1016/j.amsu.2021.103156 (PMC8689054; doi:10.1016/j.amsu.2021.103156)
Supplement: Multimedia component 1 [file mmc1.docx]

| **Clavien-Dindo Scores** | **Grade I** | **Grade II** | **Grade IIIa** | **Grade III b** | **Grade IVa** | **Grade IVb** | **Grade V** |
| --- | --- | --- | --- | --- | --- | --- | --- |
| **Hernia-Related Complications** | **(n)** | **(n)** | **(n)** | **(n)** | **(n)** | **(n)** | **(n)** |
| Death, cause unknown | 0 | 0 | 0 | 0 | 0 | 0 | 1 |
| Small bowel obstruction | 0 | 3 | 0 | 1 | 0 | 0 | 0 |
| Fascial defect | 0 | 0 | 0 | 1 | 0 | 0 | 0 |
| Postoperative urinary retention | 5 | 2 | 0 | 0 | 0 | 0 | 0 |
| Abdominal abscess | 0 | 1 | 0 | 0 | 0 | 0 | 0 |
| Abdominal adhesions | 0 | 1 | 0 | 0 | 0 | 0 | 0 |
| Abdominal distention | 1 | 0 | 0 | 0 | 0 | 0 | 0 |
| Abdominal dysesthesia | 1 | 0 | 0 | 0 | 0 | 0 | 0 |
| Constipation | 1 | 0 | 0 | 0 | 0 | 0 | 0 |
| Rash or skin irritation | 5 | 0 | 0 | 0 | 0 | 0 | 0 |
| Nausea & vomiting | 1 | 0 | 0 | 0 | 0 | 0 | 0 |
| Ileus | 5 | 0 | 0 | 0 | 0 | 0 | 0 |
| Urinary incontinence | 1 | 0 | 0 | 0 | 0 | 0 | 0 |
| Hematoma (incision site, intra-abdominal, post-procedural, subcutaneous) | 3 | 0 | 0 | 0 | 0 | 0 | 0 |
| Impaired healing | 1 | 0 | 0 | 0 | 0 | 0 | 0 |
| Pain, discomfort, tenderness (abdominal, incision site, procedural) | 34 | 0 | 0 | 0 | 0 | 0 | 0 |
| Seroma | 9 | 0 | 0 | 0 | 0 | 0 | 0 |

**Supplementary Table 1:** Clavien-Dindo classification of hernia-related complications
